# College students’ exercise experience and aggression during COVID-19: A chain mediating model

**DOI:** 10.3389/fpsyg.2022.1020679

**Published:** 2022-11-16

**Authors:** Qi Jiang

**Affiliations:** Police Academy, Shandong University of Political Science and Law, Jinan, China

**Keywords:** exercise experience, exercise well-being, exercise distress, exercise attitude, aggression

## Abstract

**Aim:**

This study aimed to explore to how exercise experience affects the aggression of college students and the mediating effects of mood and exercise attitude in COVID-19.

**Methods:**

A questionnaire survey [The Subjective Exercise Experience Scale (SEES); Profile of Mood State (POMS); Exercise Attitude Scale; and Aggression Questionnaire (AQ)] was conducted among 1,006 college students.

**Results:**

Exercise experience had a significant effect on aggression. The direct effect of exercise well-being was not significant, but indirectly affected the aggression through independent mediation and chain mediation of mood and exercise attitude. The direct effect of exercise distress was not significant, but indirectly affected the aggression through independent mediation and chain mediation of mood and exercise attitude.

**Conclusion:**

Mood and exercise attitude are powerful factors to alleviate the impact of exercise experience on aggression during the pandemic. Actively adjusting the mood and exercise attitude from a cognitive perspective may be an effective way to promote college students’ physical exercise and reduce aggression.

## Introduction

The *Health Education Manual of Pneumonia Caused by the Novel Coronavirus* (*2019-nCov*) issued by the National Health Commission recommended that the public exercise at home during the pandemic (Yan, 2020). The *Notice on Coordinating the Prevention and Control of the Novel Coronavirus Pandemic in the Education System and Education Reform and Development* issued by the Ministry of Education pointed out that it is necessary to care about the physical and mental health of students and guide them to strengthen physical exercise (Xi, 2020). Ao et al. (2020) surveyed 4,200 primary and middle school students in Chongqing and found that the situation of physical exercise at home is not optimistic, and that students have poor exercise habits. Zhang et al. (2020) surveyed 724 college students and found that the factors that have a greater impact on home physical exercise include lack of exercise venues and facilities, lack of sports partners and sports atmosphere, and decreased interest in exercise. Zhong et al. (2020) found that closed discipline during the pandemic is likely to induce more negative emotions among athletes. The pandemic has impacted the physical exercise of the group, and their exercise experience, exercise attitude, and mood may all be affected.

Studies have shown that under the stress of the pandemic, the hostility and aggression of the public will increase (Hammett et al., 2022; Wei et al., 2022). The general aggression model (GAM) also suggests that situational changes and individual cognitive factors will trigger aggressive behaviors by changing their internal states such as emotions and moods (Abreu et al., 2021; Miles-Novelo et al., 2022). At the same time, the pandemic has brought people a sense of social adversity Zhang et al. 2017 that represents the individual’s perceptual response to experiencing continuous or recurring negative social events (Monahan et al., 2022). These negative events may include social alienation and excessive control on intimate relationships, etc. Miconi et al. (2022) believed that social adversity can positively predict the aggressive behavior of college students, and can relieve anxiety, depression, anger, and other unhealthy emotions through attacks. During the pandemic, students’ interpersonal communication was affected, they lacked a sense of group membership, and they were inevitably subject to various requirements and controls from their parents at home.

Therefore, this study explores the relationship between college students’ exercise experience, mood, exercise attitude, and aggression, to provide suggestions for college students’ physical exercise, mood adjustment, and alleviation of aggression during COVID-19 in a relatively isolated environment, and to provide experience for similar events in the future.

### Exercise experience and aggression

The pandemic has caused many changes in the lives of college students. In terms of physical exercise, home exercise is very different from typical exercise (Rogowska et al., 2020; López-Valenciano et al., 2021). For example, changes in space and enclosure, exercise programs (such as long-distance running, basketball, swimming, etc.) and exercise intensity are restricted and there is a lack of exercise partners (Zhong et al., 2020). These factors will influence the effect of exercise, the most direct of which is the exercise experience. Miller (1941) discovered early that frustration could lead to aggressive behavior. The exercise experience of college students is impaired, causing exercise frustration. According to the frustration-aggression theory, if the desired target behavior is hindered, there will be aggression (Dollard and Al, 2015). The more negative life events college students experienced, the more serious the aggressive behavior (Yu et al., 2013). Experience, an element of emotion, is closely related to aggression. When negative emotions, especially anxiety levels, increase, the individual’s aggression will also increase (Zhu et al., 2017). The COVID-19 pandemic has been an intense social pressure (Wang et al., 2022) that reduced people’s sense of control and increased their sense of powerlessness (Deng and Feng, 2022). This triggered very common negative emotions (Klimovich-Mickael et al., 2021), typified by anxiety and depression (Dugré et al., 2020; Pappa et al., 2020; Salari et al., 2020), which lead to experiences of frustration that in turn increased aggression (Yang, 2021; Deng and Feng, 2022). A large number of studies have shown that physical exercise can effectively reduce aggressive behavior, but previous studies have focused on the intensity and frequency of exercise behavior, the choice of exercise activity, self-esteem or personality, and other mediating factors (Willemse et al., 2011; Jiang and Peterson, 2012; Ren et al., 2014; Zhou and Li, 2014; Clemente et al., 2016; Park et al., 2017; Han and Zheng, 2018). Less attention is paid to the impact of exercise experience on aggression. Therefore, we assume that exercise experience has a negatively predicts on college students’ aggression (H1).

### The mediating role of exercise attitude

Attitude is the relatively persistent, stable, and consistent psychological tendency of an individual (Kwol et al., 2019). The dimension of exercise attitude includes emotional experience, cognitive factors, and behavioral intention factors (Ying et al., 2020). One of the theories closely related to attitude, the rational action theory, posits that attitude can produce changes in behavior as a potential cause. Moreover, it can indirectly predict the development and changes in individual behavior and abilities through attitude (Hill et al., 1977; Wu and Li, 2016). Attitude can often play a mediating role (More and Phillips, 2022). In the model of the relationship between aggression and cognition proposed by Berkowitz (1989), it is believed that when an individual encounters a conditioned stimulus, their negative emotional experience is awakened. If cognition evaluates this emotion as anger, an aggressive reaction may result, and attitude may play a mediating or moderating role between conditioned stimulus and aggression. Berkowitz (2003, 2012) developed his own theory that people have a network in which many psychological phenomena are closely related to each other. After any part of the network is activated, it will spread to other parts. The intensity and breadth of activation depend on the degree of correlation between specific structures. These activated emotions, cognition, and experience form a larger network, which may lead to a change in attitude and then a change in behavior. For example, during the pandemic, when people get frustrated with exercise, their exercise experience becomes worse, and this negative experience can change their exercise attitude and is likely to lead to aggression.

Other studies have shown that exercise experience can effectively predict exercise attitude, and in the formation of explicit exercise attitude, the role of the emotional experience dimension is very important (Guo and Mao, 2016). Therefore, we assume that exercise attitude plays a mediating role in the influence of college students’ exercise experience on aggression (H2).

### The mediating role of mood

Mood is a complex emotional state, which is not specific but lasts a long time (Dunegan et al., 1992). It exists in consciousness, but it does not often appear explicitly, but is experienced “in the dark” (Meng, 1989). Mood provides a specific background for mental operation and affects the execution of its function (Huang and Luo, 2004). The causes of mood are various. Adversity in life, changes in the environment, personal physical state, the quality of interpersonal relationships, and so on, may become the causes of a certain state of mood (Peng, 2019).

Mood consistency bias—a recent research hotspot—refers to the fact that mood disorders can cause individuals to over-consistently pay attention to negative information or experience, and it also emphasizes the cognitive factors of mood (Delle-Vigne et al., 2014). Individuals with a positive mood always have a preference for pleasant experiences, explanations and emotional information, and can recall more pleasant materials from memory, while individuals with a negative mood do the opposite (Niedenthal and Setterlund, 1994). According to the theory of Berkowitz (2003) and Miles-Novelo et al. (2022), the spread of negative mood through association networks is likely to stimulate individual aggressiveness.

Research shows that mood disorders are more common among young people who violate the law (Qiao and Xie, 2006), and a bad shopping experience will destroy the originally good mood in consumer behavior (Swinyard and William, 1993). Nowlis (1956) proposed that mood can be considered as an intermediate intervention variable affecting the occurrence of other mental activities, and plays a role in self-monitoring, which was supported by studies (Liang et al., 2015; Liu and Jing, 2015). Therefore, we assume that mood plays a mediating role in its influence on college students’ exercise experience on aggression (H3).

### The influence of mood on attitude

**Figure 1:**
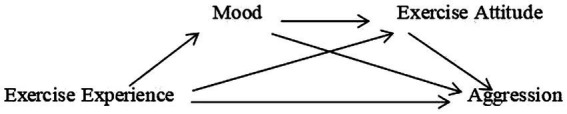
The hypothetical model of the chain mediating role.

Mood plays a vital role in the subject’s understanding of experiences and the process of further developing corresponding attitudes. Compared to indirect experiences such as learning through knowledge, mood has a priority influence in the formation of attitudes as a direct experience (Qiao, 2003). Many studies have shown that mood affects the way people process information, and can also influence the formation of attitudes (Shintaro and Morikazu, 2009; Clore and Schnall, 2005). At the same time, in terms of the influence of mood on attitude formation, Fedorikhin and Cole (2004) suggests that when the cognitive resources used for processing are limited, mood affects attitude through the process of “feeling is processing-information,” that is more about intuition or reference clues. However, when cognitive resources are not limited, the mood is carried out through the “emotional activation,” meaning that processing with a high level of construction can be more stimulating to the memory that matches the mood, and the individual mobilizes resources for adequate thinking and evaluation. Empirical research has also verified this (Norbert, 1997; Huntsinger, 2011). Therefore, we assume that exercise experience can affect the aggression of college students through the chain mediation of mood and exercise attitude (H4). In summary, this study proposes a hypothetical model diagram of chain mediation (see Figure 1).

## Materials and methods

### Participants

Using convenience and snowball sampling, a questionnaire was distributed online in September 2020, to measure the subjective exercise experience, mood state, aggression, and exercise attitude of college students from 25 provinces and municipalities.

A total of 1,217 questionnaires were collected, and the questionnaires which were too long (> 3,600 s), too short (< 300 s), or of poor quality were removed. The final number of participants was 1,006, with an effective recovery rate of 82.7%. The participants included: 711 (70.7%) males and 295 (29.3%) females; 287 (28.5%) freshmen, 319 (31.7%) sophomores, 324 (32.2%) juniors, and 76 (7.6%) seniors. Participants from urban (672, 66.8%) or rural (334, 33.2%) areas. School categories: 53 (5.3%) from higher vocational colleges, 819 (81.4%) general undergraduate colleges, and 134 (13.3%) key undergraduate colleges. Major area of study: 562 (55.9%) majored in areas related to physical exercise (e.g., physical, military, and police), and 444 (44.1%) in non-exercise related majors.

### Procedure

Data collection was collected online through WeChat software. First, the questionnaires were edited as a web link for delivery. Then, the researchers sent the link to all the college students in the Wechat address book using the Wechat private message and WeChat group, and asked the participants to complete it. Simultaneously, participants were asked to send it to the college students in their own Wechat address book to collect data scrolling. This study was approved by the ethics and law ethics committee of the Shandong University of Political Science and Law.

### Control and inspection of common method deviations

The Harman single factor test was performed on the measurement items involved (Zhou and Long, 2004). There are 22 eigenvalues greater than 1, and the variance explanation degree of the first factor is 24.63%, which is less than the critical value 40%. Therefore, there is no serious common method deviation in this study.

### Tools

#### Subjective exercise experience scale

The two subscales of exercise well-being and exercise distress have four items respectively, which are used as the measurement indicators of active exercise and negative exercise, respectively (Zhang and Mao, 2004; Dong and Zhang, 2016). Using Likert’s seven-point scoring, the higher the total score, the heavier the exercise well-being and exercise distress. An example item is, “I feel positive.” The Cronbach’s α coefficient of the exercise well-being and exercise distress scales were 0.83 and 0.87, respectively.

#### Profile of mood states scale

Revised by Zhu (1995), the short-version of POMS includes seven dimensions: tension, anger, fatigue, depression, energy, panic, and self, totaling 40 items, and employs a five-point Likert scale from 1 = not at all to 4 = extremely. The original scores of each subscale are accumulated, respectively, and the T-score of each subscale is calculated. The sum of the five negative emotion scores minus the sum of the two positive emotion scores plus 100 points is the total score of Total Mood Disturbance (TMD). The higher the TMD score, the more distressing the mood (Grove and Prapavessis, 1992). An example item is, “I am relaxed and happy.” The Cronbach’s α coefficient of the POMS was 0.91.

#### Exercise attitude scale

The physical exercise attitude is measured using the exercise attitude scale compiled by Mao (2003), that includes eight dimensions: behavior attitude, goal attitude, behavior cognition, behavior habits, behavior intention, emotional experience, behavior control, and subjective criteria, totaling 70 items with a five-point scale where the higher the total score, the more positive the attitude toward physical exercise (Yu, 2009; Zhang and Xu, 2014; Wang et al., 2015; Yang and Xu, 2016). An example item is, “I like to exercise every day.” The Cronbach’s α coefficient of the exercise attitude scale was 0.97.

#### Aggression questionnaire

The Chinese version of the Buss–Warren aggression questionnaire scale (Blurton-Jones, 1972; Li, 2008; Maxwell, 2008) was used. It includes five dimensions: physical attack, verbal attack, indirect attack, anger, and hostility, totaling 70 items with a five-point scale where the higher the total score, the more aggressive. An example item is, “I can hardly control my temper.” The Cronbach’s α coefficient of the aggression questionnaire scale was 0.93.

## Results

### Descriptive statistics of variables

The results of the descriptive statistics are shown in Table 1. Exercise well-being significantly correlates negatively with exercise distress, mood, and aggression; it is significantly correlated positively with exercise attitude. Exercise distress significantly correlates positively with mood and aggression, and negatively with exercise attitude. Mood and aggression significantly correlate positively correlated, and significantly correlate negatively with exercise attitude.

**Table 1:** Descriptive statistics of variables.

	*M*	*SD*	Exercise well-being	Exercise distress	Mood	Exercise attitude	Aggression
Exercise well-being	18.61	4.63	1				
Exercise distress	9.40	4.38	−0.40^**^	1			
Mood	118.41	22.19	−0.41^**^	058^**^	1		
Exercise attitude	256.14	36.62	0.63^**^	−0.45^**^	−0.41^**^	1	
Aggression	25.55	17.60	−0.10^**^	0.18^**^	0.20^**^	−0.18^**^	1

^*^p < 0.05, ^**^p < 0.01, and ^***^p < 0.001.

### Analysis of the mediating role of exercise attitude and mood

**Figure 2:**
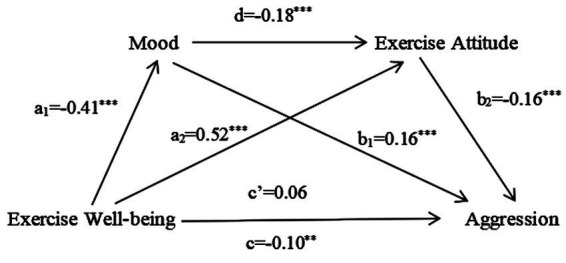
Chain mediating model test of exercise well-being. a1 predicts the regression coefficient of mood for exercise distress; a2 and d jointly predict the partial regression coefficient of exercise distress and mood; c’, b1 and b2 jointly predict the partial regression coefficient of aggression for exercise distress, mood and exercise attitude; c indicates the regression coefficient of exercise distress to aggression without mediating variables. *p<0.05, **p<0.01, and ***p<0.001.

**Figure 3:**
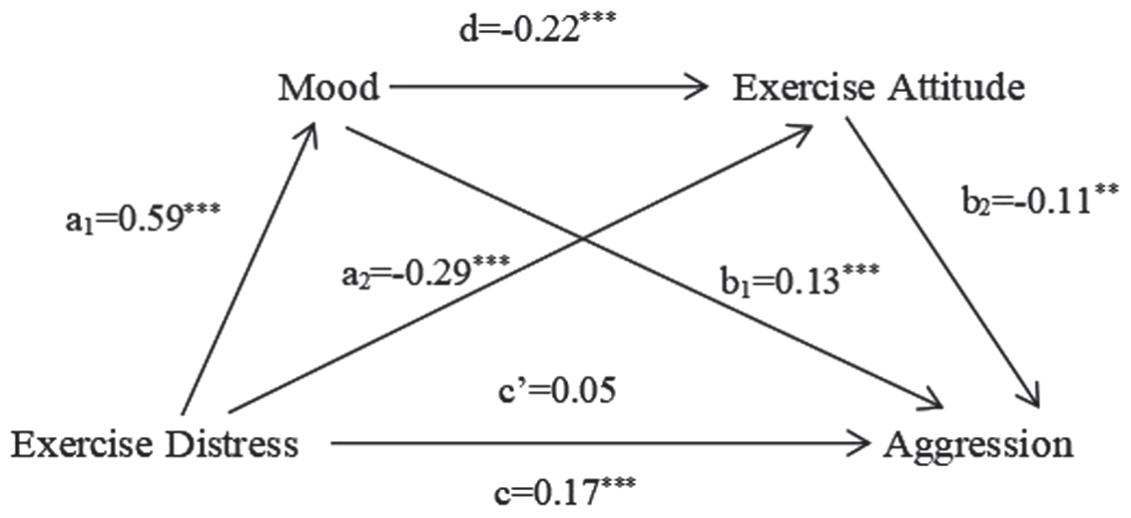
Chain mediating model test of exercise distress. a1 predicts the regression coefficient of mood for exercise distress; a2 and d jointly predict the partial regression coefficient of exercise distress and mood; c’, b1 and b2 jointly predict the partial regression coefficient of aggression for exercise distress, mood and exercise attitude; c indicates the regression coefficient of exercise distress to aggression without mediating variables. *p<0.05, **p<0.01, and ***p<0.001.

Model 6 (a chain mediating model) in the SPSS macro compiled by Hayes (2012) was applied to test the mediating effect of mood and exercise attitude in the relationship between exercise experience and aggression controlling for such variables as gender, grade, town or rural area, school category, and professional category. The results (see Figures 2, 3) show that exercise experience has a significant predictive effect on mood: exercise well-being negatively predicts mood (*B* = −0.41, *t* = −13.98, *p* < 0.001), exercise distress positively predicts mood (*B* = 0.59, *t* = 22.89, *p* < 0.001). When exercise experience and mood predict exercise attitude at the same time, the predictive effect of exercise experience is significant: exercise well-being plays a positive predictive role (*B* = 0.52, *t* = 20.45, *p* < 0.001), exercise distress plays a negative predictive role (*B* = −0.29, *t* = −8.90, *p* < 0.001), and the negative predictive effect of mood is significant (*B* = −0.18, *t* = −7.23, *p* < 0.001), (*B* = −0.22, *t* = −6.75, *p* < 0.001). When exercise experience, mood, and exercise attitude predict aggression at the same time, the direct effects of exercise well-being and exercise distress are not significant (*B* = 0.06, *t* = 1.55, *p* > 0.05), (*B* = 0.05, *t* = 1.28, *p* > 0.05), the indirect effect of this model is completely mediating. The positive predictive effect of mood is significant (*B* = 0.16, *t* = 4.78, *p* < 0.001), (*B* = 0.13, *t* = 3.32, *p* < 0.001). The negative predictive effect of exercise attitude is significant (*B* = −0.16, *t* = −3.83, *p* < 0.001), (*B* = −0.11, *t* = −3.07, *p* < 0.01).

### Mediating effects test

The analysis results of the mediation effect show (see Tables 2, 3): the bootstrap 95% CI of the total effect of exercise experience and aggression does not contain a value of 0, indicating that exercise experience has a significant predictive effect on aggression, and the effect values are −0.38 (exercise well-being) and 0.70 (exercise distress). This supports hypothesis H1. Since the direct effect of exercise experience on aggression contains a value of 0, it indicates that mood and exercise attitude have a significant and complete mediating effect between exercise experience and aggression consisting of three indirect effects with confidence intervals that do not contain a value of 0. The first indirect effect, produced by the “exercise experience→mood→aggression,” path, indicating that exercise experience has a significant mediating effect between exercise experience and aggression. The standardized effect values are −0.07 (exercise well-being) and 0.08 (exercise distress), with effect ratios of 43.75 and 66.67%, respectively. This supports hypothesis H2. The second indirect effect, produced by the “exercise experience→exercise attitude→aggression” path, indicating that exercise attitude has a significant mediating effect between exercise experience and aggression, with a standardized effect value of −0.08 (exercise well-being) and 0.03 (exercise distress), with effect ratios of 50 and 25%, respectively. This supports hypothesis H3. The third, produced by the “exercise experience→mood→exercise attitude→aggression” path, indicating that mood and exercise attitude have a significant chain mediating effect between exercise experience and aggression. The standardized effect value is −0.01 (exercise well-being) and 0.01 (exercise distress), with effect ratios of 6.25 and 8.33%, respectively. This supports hypothesis H4.

**Table 2:** Decomposition table of complete mediating effects of exercise well-being.

Influence path	Effect value	Boot Standard error	Boot CI Lower limit	Boot CI Upper limit	Effect ratio
Total indirect effect	−0.16	0.03	−0.22	−0.11	100%
Exercise well-being→mood→aggression	−0.07	0.02	−0.10	−0.04	43.75%
Exercise well-being→exercise attitude→aggression	−0.08	0.02	−0.13	−0.04	50%
Exercise well-being→mood→exercise attitude→aggression	−0.01	0.01	−0.02	−0.01	6.25%

**Table 3:** Decomposition table of complete mediating effects of exercise distress.

Influence path	Effect value	Boot Standard error	Boot CI Lower limit	Boot CI Upper limit	Effect ratio
Total indirect effect	0.12	0.03	0.07	0.17	100%
Exercise distress→mood→aggression	0.08	0.02	0.03	0.12	66.67%
Exercise distress→exercise attitude→aggression	0.03	0.01	0.01	0.06	25%
Exercise distress→mood→exercise attitude→aggression	0.01	0.01	0.01	0.03	8.33%

## Discussion

### The mediating role of mood between exercise experience and aggression

One of the goals of this study is to explore the mediating effect of mood, and the result is significant, which verifies Hypothesis 3 (H3): exercise experience can affect aggression through the mediating effect of mood. This study found that the inhibitory effect of exercise well-being through mood on aggression, and the reinforcement effect of exercise distress on aggression through mood, with effect ratios of 43.75 and 66.67%, respectively. According to the mood consistency hypothesis, individuals with a positive mood will have a preference for pleasant perception, attention, interpretation, and judgment of emotional information, and can also recall more pleasant memories, but the situation is just the opposite for individuals with a negative mood (Niedenthal and Setterlund, 1994). The results of this study are compatible with the mood consistency hypothesis.

The second goal of this study is to explore the direct effect of exercise experience on aggression. It was found that when mediating variables are added, the direct effect is not significant and Hypothesis 1 (H1) has not been verified. The working process of mood is mostly unconscious (Qiao, 2003; Christopher et al., 2005), while emotional experience is a conscious state. Therefore, the explanation for this result is that aggressive behavior is not approved by society, so whether exercise brings well-being or distress, they are not allowed to transform into aggression. Although the direct effect of the conscious level of exercise experience is limited, it can be effected through the unconscious channel of influencing the mood, that is, through the path of the mood to affect aggression.

### The mediating role of exercise attitude between exercise experience and aggression

The third goal of this study is to explore the mediating role of exercise attitudes. It was found that exercise experience can affect aggression through exercise attitudes, and Hypothesis 2 (H2) has been verified. Exercise well-being can contribute to a positive attitude towards exercise, thereby reducing aggression; exercise distress can make exercise attitudes become negative, thereby enhancing aggression. This study found that exercise well-being through exercise attitude accounted for 50% of the inhibitory effect on aggression, and exercise distress through exercise attitude accounted for 25% of the strengthening effect on aggression, which is contrary to the mediating effect of mood in the above discussion. The reason may be that mood mostly operates at an unconscious level, and attitude is a clear judgment of the level of consciousness. Individual consciousness needs to conform to social norms. Individuals should be more inclined to reduce aggression at the conscious level.

In addition, the attitudes involved in this study are explicit attitudes, which are different from the unconscious path of the mood. Cognitive evaluation can play an intermediary role through explicit and conscious forms. At the same time, this is also in line with the “emotional enlightenment” model of attitude (Clore et al., 2001), that is, emotional experience itself can determine attitude, and individuals often understand their own attitudes towards objective information based on the heuristics of direct emotional experience.

### The chain effect of exercise attitude and mood between exercise experience and aggression

The fourth goal of this study is to explore the chain mediating role of exercise attitude and mood between exercise experience and aggression, and Hypothesis 4 (H2) has also been verified. This study found that exercise experience can influence aggression through mood and exercise attitude and also influence aggression through the chain mediation of mood and exercise attitude. This involves the influence of mood on attitude and cognition. One of the manifestations of mood that is different from emotion is that mood is biased toward cognition (Davidson, 1994), its adjustment strategies are more focused on cognitive processes, such as positive or negative self-talk. This “mood inspired” model (Forgas, 1995) is consistent with the chain path effect of this research.

In addition, Smith (1998) believes that processing paths with a high level of construction will activate memories that match the mood, and individuals will mobilize resources for adequate thinking and evaluation. This study found consistency, that is, the chain mediation effect of exercise well-being accounted for 6.25%, which was lower than the effect of exercise distress accounted for 8.33%. As a negative emotional experience, exercise distress is more likely to affect exercise attitudes through negative moods, thereby increasing the possibility of aggression, but the path to exercise well-being is relatively weak.

### Limitations

First of all, this study did not take into account the differences that physical exercise might have depending on the type of sport practiced prior to the restrictions. Future studies can explore whether significant (or not significant) changes in the type and intensity of training have an impact on the mood and aggression of participants.

Secondly, in terms of measurement instruments, the widely used Sport Performance Psychological Inventory (IPED, Hernández-Mendo, 2006; Hernández-Mendo et al., 2014) includes dimensions, such as attitude control, positive coping control, and negative coping control, which are closely related to variables such as exercise attitude and aggression in this study. Future studies can use it to obtain more valuable conclusions.

## Conclusion

Exercise experience has a negative predictive effect on the aggression of college students; exercise attitude plays a mediating role in the impact of college students’ exercise experience on aggression; mood plays a mediating role in the impact of college students’ exercise experience on aggression; the exercise experience can influence the aggressiveness of college students through the chain mediation of mood and exercise attitude.

## Data availability statement

The raw data supporting the conclusions of this article will be made available by the authors, without undue reservation.

## Ethics statement

The studies involving human participants were reviewed and approved by Shandong University of Political Science and Law Ethics Committee. The patients/participants provided their written informed consent to participate in this study.

## Author contributions

The author confirms being the sole contributor of this work and has approved it for publication.

## Funding

This research was funded by Special subject of innovative Literacy of Educational Scientific Planning in Shandong Province grant number (2022CYB203).

## Conflict of interest

The author declares that the research was conducted in the absence of any commercial or financial relationships that could be construed as a potential conflict of interest.

## Publisher’s note

All claims expressed in this article are solely those of the authors and do not necessarily represent those of their affiliated organizations, or those of the publisher, the editors and the reviewers. Any product that may be evaluated in this article, or claim that may be made by its manufacturer, is not guaranteed or endorsed by the publisher.
